# A Computational Approach for Graphene Doped with N,P,B Structures as Possible Electrode Materials for Potassium Ion Batteries (PIBs): A DFT Investigation

**DOI:** 10.3390/mi16070735

**Published:** 2025-06-23

**Authors:** A. Ahmad, A. A. M. Abahussain, M. H. Nazir, S. Z. J. Zaidi

**Affiliations:** 1 Institute of Chemical Engineering and Technology, University of the Punjab, Lahore 54590, Pakistan; ranaayazahmad00@gmail.com; 2 Department of Chemical Engineering, King Saud University, P.O. Box 800, Riyadh 11451, Saudi Arabia; 3 Faculty of Computing Engineering and Sciences, University of South Wales, Pontypridd CF37 1DL, UK

**Keywords:** graphene, doping, potassium ion batteries, gibbs free energy, electronic properties

## Abstract

Although lithium-ion batteries are considered an ideal postulant for renewable energy harvesting, storage and applications, these batteries show promising performance; however, at the same time, these harvesting devices suffer from some major limitations, including scarce lithium resources, high cost, toxicity and safety concerns. Potassium ion batteries (PIBs) can be proven a favorable alternative to metal ion batteries because of their widespread potassium reserves, low costs and enhanced protection against sparks. In this study, DFT simulations were employed using the B3LYP/6-311^++^g(d p) method to explore the application of graphene and its doped variants (N,B,P-graphene) as potential anode materials for PIBs. Various key parameters such as adsorption energy, Gibbs free energy, molecular orbital energies, non-covalent interactions, cell voltage, electron density distribution and density of states were computed as a means to evaluate the suitability of materials for PIB applications. Among the four structures, nitrogen- and phosphorus-doped graphene exhibited negative Gibbs free energy values of −0.020056 and −0.021117 hartree, indicating the thermodynamic favorability of charge transfer processes. Doping graphene with nitrogen and phosphorus decreases the HOMO-LUMO gap energy, facilitating efficient ion storage and charge transport. The doping of nitrogen and phosphorus increases the cell voltage from −1.05 V to 0.54 V and 0.57 V, respectively, while boron doping decreases the cell voltage. The cell voltage produced by graphene and its doped variants in potassium ion batteries has the following order: P-graphene (0.57 V) > N-graphene (0.54 V) > graphene (−1.05 V) > B-graphene (−1.54 V). This study illustrates how nitrogen- and phosphorus-doped graphene can be used as a propitious anode electrode for PIBs.

## 1. Introduction

The development of renewable and large-scale energy harvesting technologies is crucial for the global transition towards renewable energy resources [1]. The invention of lithium-ion batteries (LIBs) led them to dominate the energy storage landscape and undergo exponential growth. Although LIBs are considered an ideal candidate and show promising performance, they suffer from some major limitations, including scarce lithium resources, high costs, toxicity and safety concerns [2]. Other metal alkaline batteries, such as sodium ion batteries (SIBs), have caught the attention of researchers in the past few years [3]. Although SIBs are less expensive and utilize abundant sodium resources, they face challenges, including large ionic radii and lower energy densities. These limitations make them an undesirable candidate for practical applications [4].

Potassium ion batteries (PIBs) have emerged as a promising alternative to traditional and well-established LIB technology. This is because of several key advantages, such as their low costs and natural abundance of potassium, lower redox potential (close to lithium-ions), high energy density and fast ion transport kinetics [5,6]. According to molecular dynamics simulations, the diffusion coefficient of the K ion is three times higher than the Li ion. All these features make KIBs a more sustainable and safe option for large-scale energy storage systems [7].

The major challenge to the development of PIBs is employing a suitable anode electrode material. Finding an appropriate material is crucial for determining the energy density, capacity, stability and conductivity of anode electrode co-doping strategies, e.g., N,P- or N,S-doped graphene, which can lead to synergistic effects, such as improving adsorption and electronic properties beyond what is achievable with single dopants, leading to the enhanced potassium ion storage performance discussed [8]. Various anode materials, including metal oxides, alloys and carbon-based materials, have been explored for PIBs. Each of these faces its own set of limitations. Alloy-type materials offer excellent conductivity but suffer from expansions in volume. Metal oxides, known for their superior capacity, suffer from lower electrical conductivity, large volume expansions and structural degradation [9].

Graphene, a nanomaterial, offers significant desirable properties such as excellent electrical conductivity, a defined surface area, superior electrochemical performance and good structural and mechanical strength [10]. Because of these properties, graphene has attracted the attention of researchers as a promising anode material for different metal ion batteries. Although graphene exhibits promising properties, pristine-phased graphene faces some challenges, such as low cell voltage (V_cell_), theoretical capacity and structural instability. Taking these limitations into consideration, it is necessary to modify the graphene structure for enhanced electrochemical performance. Graphene’s structure can be modified by doping with heteroatoms such as nitrogen (N), phosphorus (P) and boron (B). These dopants improve the electronic properties and electrochemical performance of graphene by providing additional active sites for K ion storage [11,12].

The density functional theory (DFT) is a powerful quantum chemical properties calculation tool that is used to study the electronic structures and different energies of materials. In this research work, DFT simulations were employed to investigate the potential of graphene and its doped variants with N, B and P as potential materials for an anode electrode for potassium ion batteries. By investigating key parameters such as adsorption energy, Gibbs free energy, molecular orbitals energies, non-covalent interaction, cell voltage, electron density distribution and density of states were all computed. Valuable insights into the role of doping of chemical variants in enhancing the electrochemical properties of carbon-based structures is a fascinating platform that can set out new directions in energy storage, as reported elsewhere [13,14,15,16].

## 2. Computational Details

The General Atomic and Molecular Electronic Structure System (GAMESS) software (version R1 2024) was used to carry out quantum chemical calculations [17]. In addition, Avagadro, (version 2.0), an advanced molecular editor and visualizer, was used for building geometries and the visualization of molecular orbitals [18]. Multiwfn (version 3.7) a multifunctional tool for wavefunction analysis, was utilized to generate DOS plots, study non-covalent interactions (NCIs), and electron density distribution charts [19]. The B3LYP/6-311^++^g(d p) method was used to optimize structures, the calculation of energy and electronic properties. B88&HFX was utilized as an exchange functional, and LYP88&VWN5 was used as a correlation functional for computational parameters. The DFT threshold was set as 0.958 × 10^−9^, and the grid exchange threshold was set as 0.300 × 10^−3^. In order to compute the energies of adsorption (Ead) for K @graphene and its doped variants, the following equation was employed [20]:

E_ad_= E_opt_(K@graphene) − E_opt_(graphene) − E_opt_(K)

Here, E_opt_(graphene) is the energy of the optimized graphene structure; E_opt_(K@graphene) is the energy of K ions attached to graphene’s surface; and E_opt_(K) is the energy of isolated potassium ions/atoms.

## 3. Results and Discussion

### 3.1. Structure Enhancement and Geometry Optimization

**Figure 1:**
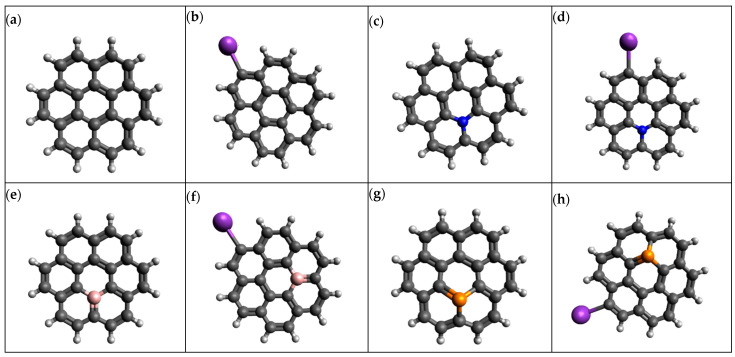
Optimized geometries of (**a**) graphene, (**b**) K@graphene, (**c**) N-graphene, (**d**) K@N-graphene, (**e**) B-graphene, (**f**) K@B-graphene, (**g**) P-graphene and (**h**) K@P-graphene.

In this study, graphene is modeled as a polycyclic arene-based coronene (C_24_H_12_) species with 24 carbon atoms at the molecular structure level, and to avoid the dangling of bonds, 12 hydrogen atoms were attached to the edges. Previous studies have demonstrated that C_24_H_12_ can be modeled as a polycyclic arene-based coronene molecule, which is termed a promising molecule to act as a prototype [21,22,23]. Figure 1 represents the structure of the polycyclic arene-based coronene molecule, which is modeled as C_24_H_12_. The doped variants of graphene were built by replacing the carbon atoms with heteroatoms such as nitrogen (N), phosphorus (P) and boron (B). The inebriated C_24_H_12_, thus, consists of 23 carbon atoms, 12 hydrogen atoms and 1 heteroatom (N, B and P). The K atom was replaced with one hydrogen atom on the edges of the graphene structure in order to create K@graphene structures. All the structures were created in the Avagadro molecular editor.

**Figure 2:**
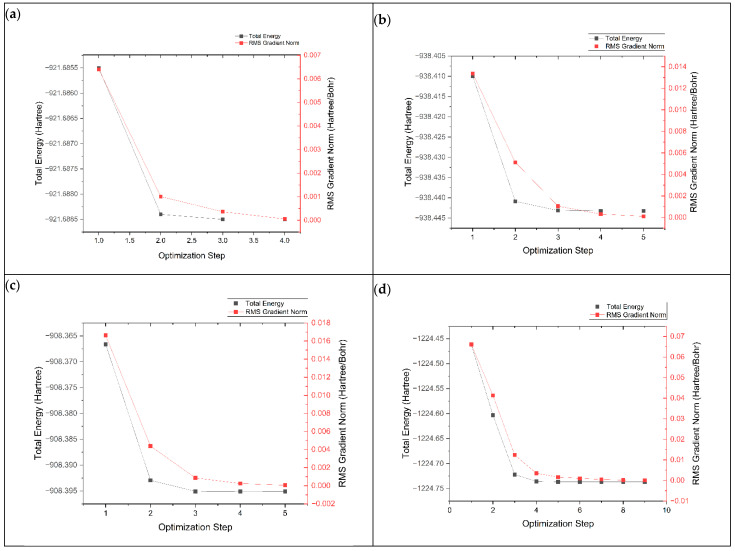
Geometry optimization plots of (**a**) graphene, (**b**) N-graphene, (**c**) B-graphene and (**d**) P-graphene.

**Figure 3:**
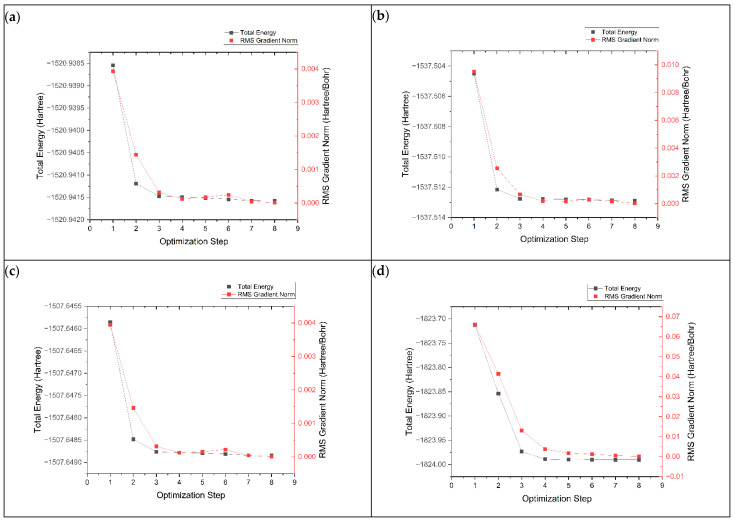
Geometry optimization plots of (**a**) K@graphene, (**b**) K@N-graphene, (**c**) K@B-graphene and (**d**) K@P-graphene.

The geometry optimization process is important for finding the most stable electronic structure with the lowest potential energy. This is performed by adjusting the atoms in the structure to minimize their energy. The geometry optimization process of graphene, K@graphene, N-graphene, K@N-graphene, B-graphene, K@B-graphene, P-graphene and K@P-graphene involves the convergence of energy and root mean square (RMS) values across optimization steps. In Figure 2, plot (a) represents the optimization process of pristine-phased graphene. It can be observed that the optimization process is straightforward and simple and requires four steps to achieve convergence. The energy states exhibit a smooth decreasing trend with fewer local minima. In the case of N- and B-doped graphene (plots b and c), the optimization process becomes more complex. It requires around five steps to achieve convergence. The addition of nitrogen and boron atoms to the graphene structure creates new electronic states. It also creates local distortions in the graphene lattice, causing a marginally more rugged energy landscape. P-graphene (plot d) shows the most complex geometry optimization process among all other structures, requiring nine steps for convergence. The main reason for this complexity in geometry optimization is that the electronic structure of graphene is more intensely altered. Furthermore, the phosphorus atom has a defined size compared to the C, N and B atoms, creating a more significant distortion in the graphene structure. This distortion causes more stress and strain in the structure, making the geometry optimization process more complex. Figure 3 shows the geometry optimization plots of pristine-phased grapheme along with its doped variants after the adsorption of K ions.

### 3.2. Adsorption Energy Calculations

Adsorption energy is the energy associated with the interaction of potassium ions with the surface of anode material. To study the adsorption energy of potassium ions on the graphene and its doped variants, the following equation can be used to calculate adsorption energies.

E_ad_ = E_opt_(K@graphene) − E_opt_(graphene) − E_opt_(K)

According to the above equation, the more highly negative the value of adsorption energy is, the stronger the interaction is between the K ion and graphene and its doped variants. The strong interaction between the K ion and the surface of the anode material can lead to better structural stability and enhanced performance of anode materials for battery applications. And a weaker interaction between the K ion and anode material can compromise the anode’s structural stability and electrochemical performance. Table 1 shows the adsorption energies of potassium ions over the surfaces of graphene-based structures.

Graphene, B-graphene and P-graphene exhibit absorption energies of −2.34742, −2.34810 and −2.34778 hartree, respectively. These higher values of absorption energies indicate strong binding and excellent structural stability, making the respective structures potential candidates for PIBs, offering durability and reliability. This can compromise the structural durability and stability of anode materials. Therefore, boron and phosphorus doping are beneficial for the structural stability and performance of PIBs.

### 3.3. Cell Voltage and Gibbs Free Energy Change

Cell voltage (V_cell_) and changes in Gibbs free energy (ΔG) are critical parameters for determining the suitability of anode materials in potassium ion batteries. A negative value of ΔG indicates the thermodynamic favorability and spontaneity of K ions in insertion and disinsertion processes. The cell voltage (V_cell_) acts as a driving force for K ion insertion and disinsertion within the anode structure. The positive value of cell voltage (V_cell_) defines efficient ion storage and release. If graphene-based structures are used as the anode material for PIBs, the following reactions occur at the anode and cathode: K@Graphene ↔ K+ @Graphene + e^−^ (at the anode) and K^+^ + e^−^ ↔ K (at the cathode). The equation below shows the overall reaction:

K^+^ + K@CNC ↔ K^+^@CNC + K

Nernst expressions can be employed to compute the cell voltage as follows:

V_cell_ = ΔG/zF

Here, ΔG is the change in Gibbs free energy, F is the Faraday constant and z is the number of electrons transferred. ΔG can be computed as follows:

ΔG = ΔE + PΔV − TΔS

The contribution of change in volume and entropy to ΔG is negligible, so after omitting these terms, the final equation can be written as follows:

ΔE_cell_~ΔG_cell_ = E(K) + E(K^+^@graphene) − E(K^+^) − E(K@graphene)

Table 2 shows the values of computed ΔG and Vcell values for different graphene structures.

In the case of graphene, the positive value of ΔG = 0.038439 hartree indicates that the insertion of K ions is not thermodynamically favorable under standard conditions. The negative value of Vcell = −1.05 V indicates the lack of driving force for K ion insertion and disinsertion in graphene’s structure. As such, here, the conclusion can be drawn that graphene without doping is not a suitable candidate for anode material.

N-graphene exhibits ΔG = −0.020056 hartree and V_cell_ = 0.54 V. The negative value of ΔG indicates that the ion storage process is energetically spontaneous, which is advantageous for battery operations. The positive value of V_cell_ account for efficient ion storage and release, enhancing the overall battery performance. Thus, it can be inferred that doping the graphene structure with a nitrogen atom makes it suitable for an anode electrode for PIBs.

**Figure 4:**
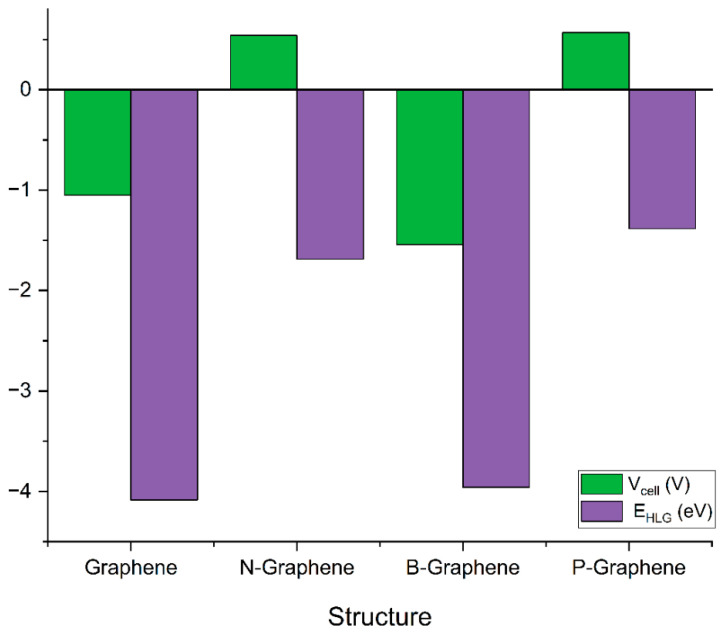
Gibbs free energy change (ΔG) and cell voltage (V_cell_) values for different graphene structures.

Doping graphene with boron exhibits ΔG = 0.056729 hatree and V_cell_ = −1.54 V. The positive value of ΔG indicates that the material is not suitable for K ion storage. The higher negative values of cell voltage accounts for the poor performance of B-graphene as an anode electrode for potassium ion batteries. On the other hand, phosphorus-doped graphene shows ΔG = −0.021117 hatree and V_cell_ = −0.57 V. The negative value of ΔG and positive value of V_cell_ indicate the feasibility of B-graphene for its application as an anode electrode for PIBs as shown in Figure 4. Thus, it can be concluded that doping graphene with nitrogen and phosphorus enhances its electronic properties and electrochemical performance. On the other hand, simple graphene and its B-doped variant are suitable for PIBs.

### 3.4. Molecular Orbital Analysis and Energies

**Figure 5:**
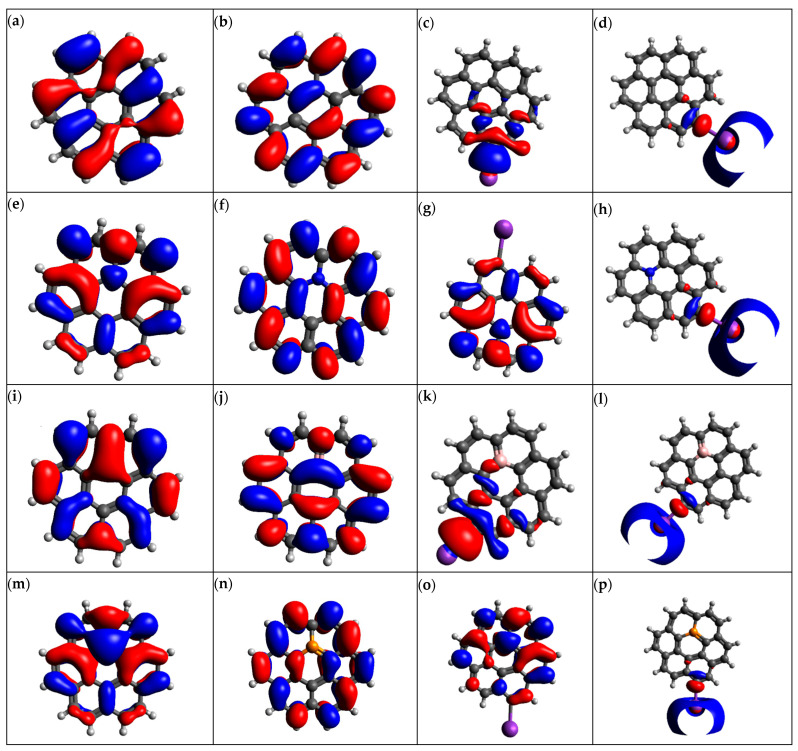
Molecular orbitals of (**a**) graphene HOMO, (**b**) graphene LUMO, (**c**) K@graphene HOMO, (**d**) K@graphene LUMO, (**e**) N-graphene HOMO, (**f**) N-graphene LUMO, (**g**) K@N-graphene HOMO, (**h**) K@N-graphene LUMO, (**i**) B-graphene HOMO, (**j**) B-graphene LUMO, (**k**) K@B-graphene HOMO, (**l**) K@B-graphene LUMO, (**m**) P-graphene HOMO, (**n**) P-graphene LUMO, (**o**) K@P-graphene HOMO and (**p**) K@P-graphene LUMO.

In the potassium ion battery system, parameters such as the highest occupied molecular orbital energy (E_HOMO_), lowest unoccupied molecular orbital energy (E_LUMO_) and homo lumo gap energy (E_HLG_) are critical in determining the electronic properties and reactivity of anode material. The molecular orbitals for graphene and its doped variant are depicted in Figure 5. E_HOMO_ is the energy associated with the removal of an electron, and E_LOMO_ is the energy required to add an electron. Material reactivity can be predicted by E_HLG_. If the HOMO-LUMO energy gap is smaller, a smaller amount of energy is required to facilitate the electron transfer process. The computed values of molecular orbital energies of graphene structures before and after the adsorption of potassium ions are given in Table 3 and Table 4. Pristine graphene has an E_HLG_ of −4.0854 eV. This higher value of the HOMO-LUMO gap indicates the reduced reactivity of graphene with potassium ions.

This means that graphene will not interact with K ions efficiently, making graphene undesirable as an anode material for PIBs. Doping graphene with nitrogen reduces the HOMO-LUMO gap to −1.6860 eV. This smaller energy gap indicates higher reactivity and an efficient charge transfer process, making N-graphene a suitable candidate for the anode electrode of PIBs. Boron-doped graphene (B-graphene) exhibits an E_HLG_ of −3.9576 eV, which is almost similar to the graphene HOMO-LUMO gap. The higher value of the HOMO-LUMO gap indicates that the interaction and charge transfer of B-graphene with K ions is not efficient. P-graphene shows the smallest value of the HOMO-LUMO gap of −1.3832 eV among all graphene structures. This is because adding phosphorus to graphene structures introduces new energy levels. It also alters the charge distribution and hybridization, leading to the enhanced reactivity of the material. This smaller HLG makes P-doped graphene an ideal candidate for the anode material of PIBs.

The adsorption of K ions on graphene-based structures alters their electronic properties significantly. In the case of K@Graphene, the energy of HLG is 2.5057 eV, indicating its increased reactivity. K@N-graphene shows a reduced E_HLG_ of −0.5397 eV. Similarly, B- and P-doped graphene also show reduced HLG energies of −2.5068 and −0.4719 eV, respectively. The reduction in HLG after the adsorption of K ion on the graphene-based structures occurs because the donation of electrons by potassium ions facilitates the charge transfer process and reduces the graphene.

The LUMO energy is lowered because some unoccupied orbitals present in graphene are filled, and in this way, HLG becomes narrow. The electron distribution and bond structures are modified because of the distortion in the graphene lattice due to the induced dipole moments of potassium ions.

### 3.5. Electronic Properties

Table 5 represents the electronic properties of graphene and its doped variants. These electronic properties include chemical potential (*μ*), chemical hardness (*η*), chemical softness (*s*) and the electrophilcity index (*ω*).

Chemical potential (*μ*) is the measure of the tendency of a material to lose electrons. N-graphene and P-graphene show fewer negative values of *μ* compared to graphene and B-graphene. This means that N- and P-graphene have a higher reactivity and higher tendency to donate electrons to potassium, making them favorable candidates for PIB anodes. The more negative values there are of *μ* for graphene and B-graphene, the more stable their electronic configuration is and the lower their reactivity. Chemical hardness (*η*) and chemical softness (*s*) are inverse to each other. According to the computed results, graphene and B-graphene exhibit higher values of chemical hardness of 2.0428 eV and 1.97975 eV, respectively. N- and P-graphene are softer than other graphene structures, indicating their better ability to store ions. This thing makes them promising candidates for anodes.

The electrophilcity index (*ω*) quantifies the ability of a material to accept the electrons from a donor. Among all four structures, phosphorus-doped graphene has the highest value of *ω* = 4.1346 eV. After this, N-graphene has the highest value of the electrophilcity index (*ω*) = 3.6454 eV. It can be concluded that phosphorus and nitrogen doping make graphene efficient for accepting electrons from potassium ions. Graphene and B-graphene exhibit a lower electrophilcity index (*ω*), indicating their reduced ability to accept electrons and interact with potassium ions, making them the least desirable candidates for anode electrodes for PIBs.

### 3.6. Density of State (DOS) Plots

**Figure 6:**
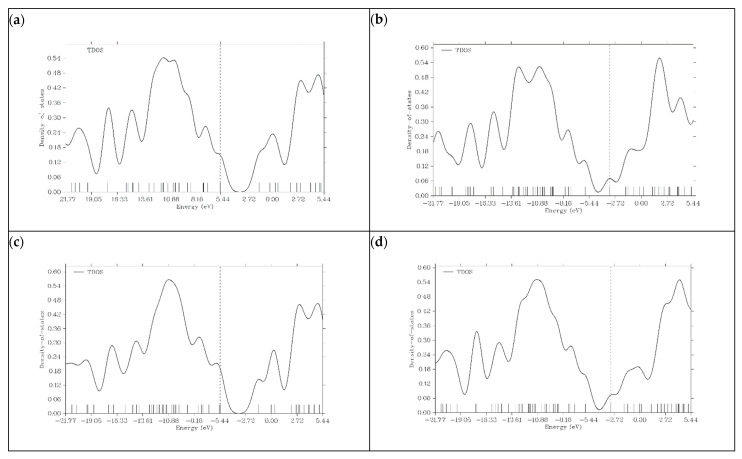
Density of state (DOS) plots for (**a**) graphene, (**b**) N-graphene, (**c**) B-graphene and (**d**) P-graphene.

Density of state (DOS) plots have been proven to be instrumental in obtaining information about the electronic structures of a particular material. DOS represents the energy states available for electrons to occupy in a base structure. Figure 6 shows the density of state (DOS) plots for graphene and its doped variants [24].

**Figure 7:**
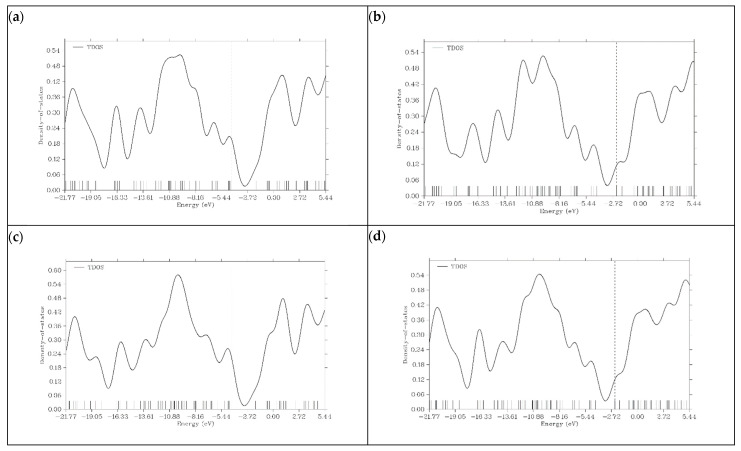
Density of state (DOS) plots for (**a**) K@graphene, (**b**) K@N-graphene, (**c**) K@B-graphene and (**d**) K@P-graphene.

In the case of graphene, DOS represents a typical electronic structure. The significant gap near the Fermi level indicates the semi-metal behavior of graphene. The limited reactivity and poor conductivity of graphene can be predicted by the absence of states at the Fermi level. Taking all these results into consideration, we can say that graphene, without doping, is not a suitable material for PIB anodes. N- and P-doped graphene shows a significant increase in its density of states near the Fermi level. The conductivity and reactivity of both materials are enhanced. This is because doping introduces additional states within the bandgap. The HOMO energy levels of N- and P-doped graphene are −3.3222 and −3.0818 eV, respectively, which can be seen on the dashed lines present in their DOS plots. Due to the P and N atoms present in the graphene structure, graphene donates extra electrons during the charge transfer process, and N- and P-doped graphene act as n-type semiconductors. Boron doping in graphene does not show an increase in electronic states around the Fermi level like P- and N-doped variants do. The HOMO energy level of B-graphene present in the DOS plot is −5.4197 eV, which indicates p-type semiconductor behavior because of its tendency to accept electrons. This behavior is undesirable for efficient battery operation. In all graphene-based structures, after the addition of K ions on the surface, the number of electronic states near the Fermi level increases. This is because potassium is an alkali metal, and it shows a tendency to lose electrons. Figure 7 illustrates the DOS plots of graphene and its doped variants after the adsorption of K ions.

### 3.7. Non-Covalent Interaction Analysis

**Figure 8:**
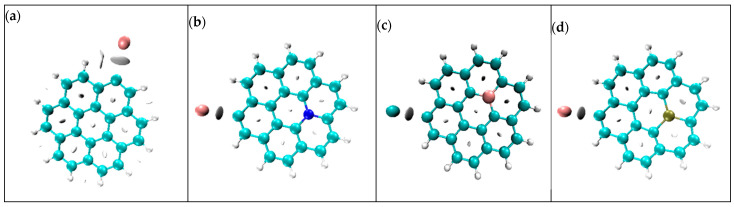
Visualization of non-covalent interactions of (**a**) K@graphene, (**b**) K@N-graphene, (**c**) K@B-graphene and (**d**) K@P-graphene.

The visualization of non-covalent interactions is shown in Figure 8. The plots represent the interaction of potassium ions with graphene and its doped variants. These interactions are crucial for better battery performance, conductivity and the reactivity of anode material [25].

In Figure 8 plot (a) shows the interaction between graphene and potassium ions. The interaction is non-covalent and involves Van der Walls interactions. It also consists of electrostatic attractions. Plots (b) and (d) represent the interaction of K ions with N- and P-doped graphene structures. Nitrogen and phosphorus doping produces more electronic states and localized charges, leading to a stronger interaction between the ion and surface due to additional electrostatic attractions. This improves the charge storage capacity and speeds up the charge/discharge process. In the case of B-graphene, the interaction between K ions and the anode material is shown in plot (c); the interaction is strong but not as strong as N- and P-doped graphene due to limited electronic states and Van der Walls interactions.

### 3.8. Electron Density Distribution Analysis

**Figure 9:**
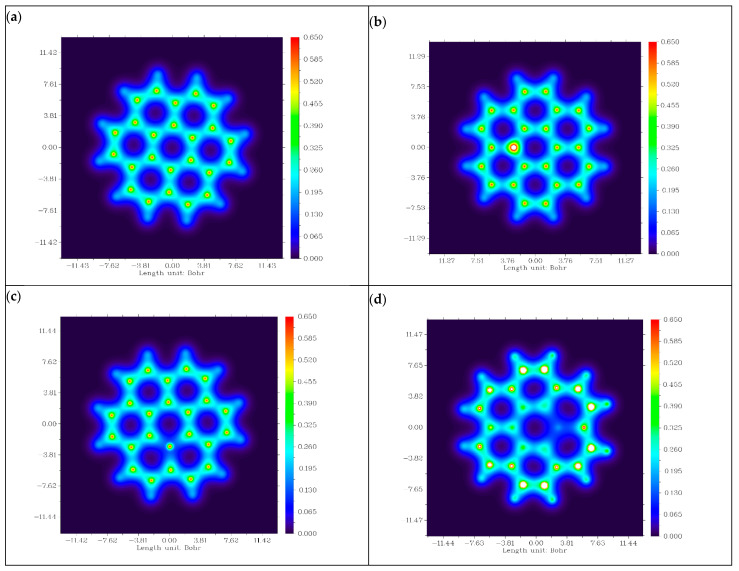
Electron density distribution charts of (**a**) graphene, (**b**) N-graphene, (**c**) B-graphene and (**d**) P-graphene.

Figure 9 illustrates the distribution of electron density along the surface of graphene and its doped variants in terms of electron density distribution charts [26]. In chart (a), a uniform electron density distribution can be observed across the hexagonal lattice structure of carbon atoms. This uniformity in electron density distribution is due to the sp2 hybridization of carbon atoms. Due to this hybridization, the presence of a delocalized π-electron system over the lattice of graphene emerges. This uniform distribution indicates the lower conductivity and reactivity of graphene as an anode material for PIBs.

Chart (b) shows the electron distribution across the surface of nitrogen-doped graphene. We observed regions with an increased electron density across the nitrogen atom. This increase in the electron density was due to the addition of a lone pair of electrons via nitrogen into the graphene structure. This increment in electron density contributes to the enhanced reactivity and conductivity of the material, making it an n-type semiconductor. For the boron-doped variant of graphene, a reduction in electron density near the boron atom can be observed. This is because boron has one less electron than carbon, creating electro-deficient sites within the structure. In this way, it behaves as a p-type semiconductor, showing a tendency to accept the electrons. In the electron density distribution chart of P-graphene, regions of increased electron density near the phosphorus atom were observed. This is because the phosphorus atom has more valance electrons than carbon atoms. This increased electron density facilitates the electron transfer process, enhancing the overall conductivity and reactivity of the material. On the basis of electron density distribution, it can be concluded that the N- and P-doped variants of graphene are favorable candidates for anode electrodes of PIBs. 

## 4. Conclusions

Despite lithium-ion batteries being an ideal candidate for energy storage and showing promising performance, they suffer from some major issues, including limited lithium resources, high costs, toxicity and safety concerns. Therefore, other metal alkaline batteries, such as sodium ion and potassium ion batteries, have caught the attention of researchers over the past few years. In this study, we investigated graphene and its three doped variants (N, B and P) as possible anode electrodes for PIBs using the computational DFT approach.

The results showed that graphene, B-graphene and P-graphene exhibit absorption energies of −2.34742, −2.34810 and −2.34778 hartree, respectively as shown in Table 6. Conversely, N-graphene showed an adsorption energy of −2.16403 hartree, indicating weaker atomic binding between K ions and the anode structure. N-graphene and P-graphene exhibited negative values of ΔG = −0.020056 and −0.021117 hartree, respectively, indicating the thermodynamic favorability of the charge transfer process. The trend of cell voltage produced by graphene and its doped variants in potassium ion batteries showed the following order: N-graphene (0.54 V) > P-graphene (0.57 V) > graphene (−1.05 V) > B-graphene (−1.54V).

## Figures and Tables

**Table 1:** Adsorption energies of K ions over the surfaces of graphene-based structures.

Structure	E_ad_ (Hartree)	E_ad_ (Kcal/mol)
Graphene	−2.34742	−1472.26
N-Graphene	−2.16403	−1357.78
B-Graphene	−2.34810	−1472.66
P-Graphene	−2.34778	−1472.48

**Table 2:** Computed values of Gibbs free energy change (ΔG) and cell voltage (V_cell_) values for different graphene structures.

Structure	ΔG (Hartree)	V_cell_ (V)
Graphene	0.038439	−1.05
N-Graphene	−0.020056	0.54
B-Graphene	0.056729	−1.54
P-Graphene	−0.021117	0.57

**Table 3:** Molecular orbital energies (E_HOMO,_ E_LUMO_ and E_HLG_) of graphene-based structures.

Structure	E_HOMO_ (eV)	E_LUMO_ (eV)	E_HLG_ (eV)
Graphene	−5.4900	−1.4045	−4.0854
N-Graphene	−3.3222	−1.6363	−1.6860
B-Graphene	−5.4197	−1.4602	−3.9576
P-Graphene	−3.0818	−1.6994	−1.3832

**Table 4:** Molecular orbital energies (E_HOMO,_ E_LUMO_ and E_HLG_) of graphene-based structures after K ion adsorption.

Structure	E_HOMO_ (eV)	E_LUMO_ (eV)	E_HLG_ (eV)
K@Graphene	−4.3492	−1.8435	−2.5057
K@N-Graphene	−2.3847	−1.8460	−0.5397
K@B-Graphene	−4.3627	−1.8538	−2.5068
K@P-Graphene	−2.3486	−1.8771	−0.4719

**Table 5:** Electronic properties of graphene and its doped variants.

Structure	*μ* (eV)	*η* (eV)	*s* (eV^−1^)	*ω* (eV)
Graphene	−3.4473	2.0428	0.4896	2.9085
N-Graphene	−2.4793	0.84295	1.1865	3.6454
B-Graphene	−3.43995	1.97975	0.5051	2.9889
P-Graphene	−2.3906	0.6912	1.4468	4.1346

**Table 6:** Electronic and thermodynamic properties of graphene and its doped variants.

Structure	E_HOMO_ (eV)	E_LUMO_ (eV)	E_HLG_ (eV)	ΔG (Hartree)	V_cell_ (V)	E_ad_ (Hartree)
Graphene	−5.4900	−1.4045	−4.0854	0.038439	−1.05	−2.34742
N-Graphene	−3.3222	−1.6363	−1.6860	−0.020056	0.54	−2.16403
B-Graphene	−5.4197	−1.4602	−3.9576	0.056729	−1.54	−2.34810
P-Graphene	−3.0818	−1.6994	−1.3832	−0.021117	0.57	−2.34778

## Data Availability

The data that support the findings of this study are available from the corresponding author upon reasonable request.
